# Supplementary material for: Content-rich biological network constructed by mining PubMed abstracts
Source: BMC Bioinformatics. 2004 Oct 8;5:147. doi: 10.1186/1471-2105-5-147 (PMC528731; doi:10.1186/1471-2105-5-147)
Supplement: Additional File 5 — The original Chilibot query results of the term "long-term potentiation (LTP)" and 22 other terms, limiting the latest references analyzed to the years 1990, 1995, 2000, and 2004. [file 1471-2105-5-147-S5.bz2 › chilibotAdditionalFile5/ltp2004/html/TRKA_PI-3K.html]

 


 **TRKA** and **PI-3K** 
  
Found 28 abstracts in PubMed,  **28 abstracts were retrieved and analyzed**.  


---

 Search Google  |
 PDF files only 
|  EDU domain only 

---

**Interactive relationship** (e.g. stimulation, inhibition, etc)

- Overall, DQ elicited the differentiation of PC12 cells through Trk A  [ **TRKA** ]  phosphorylation followed by Erk 1 2 and PI3 kinase  [ **PI-3K** ]  activation.  Ref: 14706634 Biochem Biophys Res Commun, 2004
- NGF binding to the high affinity tyrosine kinase  **TrkA**  receptors initiates second messenger signalling cascades, one of which includes the activation of phosphoinositide 3 kinase PI3 kinase  [ **PI-3K** ] .  Ref: 9252024 Brain Res, 1997
- In addition to rapid increases in the phosphorylation of phosphatidylinositol 3 kinase PI 3 kinase  [ **PI-3K** ]  and phospholipase C gamma and increased ras activity, phosphorylation of c Crk and paxillin proteins has been observed upon  **TrkA**  activation.  Ref: 10679771 J Neurosci Res, 2000
- As sensory neurones also depend on NGF for their developmental survival and endocytose and retrogradely transport the NGF  **TrkA**  receptor complex, this population of neurones may either recruit a different isoform of PI3 kinase  [ **PI-3K** ]  or utilize PI3 kinase  [ **PI-3K** ]  independent signalling pathways for these cellular functions.  Ref: 9252024 Brain Res, 1997
- As PI3 kinase  [ **PI-3K** ]  is present in both neuronal populations, this suggests that the Wortmannin sensitive isoform of PI3 kinase  [ **PI-3K** ]  p110 is essential in sympathetic neurones both for survival and for NGF  **TrkA**  receptor complex trafficking.  Ref: 9252024 Brain Res, 1997
- In this comparison, significant differences were observed in the tyrosine phosphorylation pattern of PI 3 kinase  [ **PI-3K** ]  associated polypeptides, suggesting the existence of cell type specific molecular interactions influencing  **trkA**  signaling.  Ref: 7563243 J Neurosci Res, 1995
- In summary, our data suggest that PI 3 kinase  [ **PI-3K** ]  regulates the inhibitory crosstalk between Trk A  [ **TRKA** ]  tyrosine kinase and p75 NTR dependent sphingolipid signaling pathways.  Ref: 11238738 J Neurochem, 2001

**Parallel relationship** (e.g. studied together, co-existance, homology, etc.)

- The downstream activation of phosphatidylinositol 3 kinase PI 3 kinase  [ **PI-3K** ]  is essential for the inhibition of apoptosis, although this enzyme does not bind to and is not directly activated by  **TrkA** .  Ref: 9356464 Proc Natl Acad Sci U S A, 1997
- Mutational analysis indicates that coactivation of phospholipase C gamma PLC gamma and phosphoinositide 3 kinase PI3 kinase  [ **PI-3K** ]  by specific cytoplasmic domains of  **TrkA**  is essential for triggering chemoattraction of the growth cone in an NGF gradient.  Ref: 10402200 Neuron, 1999
- Mechanistically, NGF stimulation of PC12 cells resulted in a weak or possibly indirect association between  **trkA**  and PI 3 kinase  [ **PI-3K** ] .  Ref: 7563243 J Neurosci Res, 1995
- Hence, Gab1 has a major role in connecting  **TrkA**  with PI 3 kinase  [ **PI-3K** ]  activation and for the promotion of cell survival by NGF.  Ref: 9356464 Proc Natl Acad Sci U S A, 1997
- The phosphoinositide 3 kinase PI 3 kinase  [ **PI-3K** ]  inhibitor, LY294002, reversed this inhibition suggesting that Trk A  [ **TRKA** ]  activation of PI 3 kinase  [ **PI-3K** ]  is necessary to inhibit sphingolipid signaling by p75 NTR.  Ref: 11238738 J Neurochem, 2001
- We propose that mitochondrial transport and or docking are regulated in part via NGF  **TrkA**  PI 3 kinase  [ **PI-3K** ]  signaling.  Ref: 12756280 J Exp Biol, 2003
- C6 ceramide induces  **TrkA**  phosphorylation and selective activation of the phosphatidyl inositol 3 kinase PI3 kinase  [ **PI-3K** ]  Akt pathway but not the MAPK ERK pathway.  Ref: 14614956 Neuropharmacology, 2003
- Previous studies have shown that activation of phosphatidylinositol 3 kinase PI 3 kinase  [ **PI-3K** ]  by NGF via the  **TrkA**  receptor tyrosine kinase protects PC12 cells from serum deprivation induced apoptosis.  Ref: 9202293 J Neurochem, 1997
- NGF increased  **TrkA**  phosphorylation in hippocampal neurons and provided protection that required phosphoinositol 3 phosphate PI3 kinase  [ **PI-3K** ]  activity and Akt phosphorylation.  Ref: 12453482 Neuroscience, 2002
